# Competence of birth attendants at providing emergency obstetric care under India’s JSY conditional cash transfer program for institutional delivery: an assessment using case vignettes in Madhya Pradesh province

**DOI:** 10.1186/1471-2393-14-174

**Published:** 2014-05-24

**Authors:** Sarika Chaturvedi, Sourabh Upadhyay, Ayesha De Costa

**Affiliations:** 1 Department of Public Health and Environment, R D Gardi Medical College, Ujjain, India; 2 Department of Public Health Sciences, Karolinska Institutet, Stockholm, Sweden

**Keywords:** Clinical competence, Emergency obstetric care, Conditional cash transfer, Obstetric Nursing, Maternal mortality, Midwifery, JSY, India, Vignettes

## Abstract

**Background:**

Access to emergency obstetric care by competent staff can reduce maternal mortality. India has launched the Janani Suraksha Yojana (JSY) conditional cash transfer program to promote institutional births. During implementation of the JSY, India witnessed a steep increase in the proportion of institutional deliveries-from 40% in 2004 to 73% in 2012. However, maternal mortality reduction follows a secular trend. Competent management of complications, when women deliver in facilities under the JSY, is essential for reduction in maternal mortality and therefore to a successful program outcome. We investigate, using clinical vignettes, whether birth attendants at institutions under the program are competent at providing appropriate care for obstetric complications.

**Methods:**

A facility based cross-sectional study was conducted in three districts of Madhya Pradesh (MP) province. Written case vignettes for two obstetric complications, hemorrhage and eclampsia, were administered to 233 birth attendant nurses at 73 JSY facilities. Their competence at (a) initial assessment, (b) diagnosis, and (c) making decisions on appropriate first-line care for these complications was scored.

**Results:**

The mean emergency obstetric care (EmOC) competence score was 5.4 (median = 5) on a total score of 20, and 75% of participants scored below 35% of the maximum score. The overall score, although poor, was marginally higher in respondents with Skilled Birth Attendant (SBA) training, those with general nursing and midwifery qualifications, those at higher facility levels, and those conducting >30 deliveries a month. In all, 14% of respondents were competent at assessment, 58% were competent at making a correct clinical diagnosis, and 20% were competent at providing first-line care.

**Conclusions:**

Birth attendants in the JSY facilities have low competence at EmOC provision. Hence, births in the JSY program cannot be considered to have access to competent EmOC. Urgent efforts are required to effectively increase the competence of birth attendants at managing obstetric complications in order to translate large gains in coverage of institutional delivery services under JSY into reductions in maternal mortality in Madhya Pradesh, India.

## Background

Access to emergency obstetric care (EmOC) can significantly reduce maternal mortality and morbidity. Skilled attendance at birth is associated with reduction in maternal mortality, however the relationship is weak in developing countries [1] and especially in countries where the maternal mortality ratio (MMR) exceeds 200 per 100,000 live births [2]. Low MMR associated with skilled birth attendance is largely due to identification and treatment of complications in the context of functioning health systems in high income countries [2]. Most maternal deaths occur during labor, delivery, or the first 24 hours postpartum, and most life-threatening obstetric complications cannot be predicted or prevented. When complications occur, a timely diagnosis and appropriate intervention, both of which require considerable skill, can prevent death or morbidity. The location of women when they deliver, the person or persons attending to them, and how quickly they can be transported to referral-level care are critical to the success of life-saving interventions [3]. Thus an effective intra-partum care strategy is a priority to reduce maternal mortality [4].Evidence shows that the best intra-partum care strategy is likely to be one in which women routinely choose to deliver in a health center, with midwives as the main providers, and other attendants working with them in a team. Underlying this strategy, however, are important principles of safety, early detection, and management of complications, including life-threatening ones [4]. Depending on the level of the facility, the management of complications would include first-line care prior to referral, or more complete management, including caesarean section, at higher-level facilities.

Ensuring that facilities can provide adequate EmOC involves strengthening the supply side of the health system through upgrading physical infrastructure, the recruiting and training of staff to deliver care, ensuring adequate medical supplies and equipment, and having a functioning referral system. Maternal health programs in India, during the 90s and up until 2004, focused on strengthening institutional capacity. However utilization of health facilities for obstetric care remained low. About 60% of births continued to occur outside health facilities [5]. To reduce financial-access barriers to intra-partum care at a health facility, India instituted the *Janani Suraksha Yojana* (JSY) program in 2005 [6]. The program is a cash transfer to mothers when they deliver in a health facility. This program successfully raised institutional delivery proportions to 73% in 2012 [7]. There have been 70 million beneficiaries of JSY by end of 2013 [8]. However, despite the steep rise in institutional delivery during the JSY implementation period, MMR decline follows a secular trend. MMR dropped from 254 (95% CI: 239–269) to178 (95% CI: 166–191) per 100,000 births between 2004-06 [9] and 2010-12 [7]. Lim et al. [10], Randive et al. [11], and De Costa et al. [12], were unable to detect a significant reduction in MMR associated with JSY uptake. These evaluations of the program suggest possible gaps in quality of care at institutions as a reason for its limited success. However, there are few reported empirical assessments of quality of care across JSY program facilities.

The JSY program rationale assumes institutional birth promotion as an essential step to increase access to EmOC, so leading to maternal mortality reduction. Analysis of a quasi-experimental study in Bangladesh [13] and of a cohort study in rural Maharashtra, India [14], suggests that EmOC can be effective at maternal mortality reduction even when all deliveries are not conducted by skilled birth attendants. This is provided that obstetric complications are recognized correctly by birth attendants, and women are referred in time to facilities with good quality, emergency obstetric services. Both these examples highlight the importance of competence in identification and appropriate management of complications in the reduction of maternal mortality. Hence, to investigate the paradox of persisting maternal mortality, despite steep increases in institutional deliveries during the JSY, it is important to investigate whether the care at institutions under the program, particularly for obstetric complications seen in this context, is appropriate to save lives.

As nurses attend the majority of deliveries under the JSY cash transfer program, we studied, using case vignettes, their competence at (a) initially assessing specific obstetric complications, (b) diagnosing the complication, and (c) making decisions on appropriate first-line care. This study addresses a critical aspect of quality of care under the program - the competence of staff to recognize and manage complications adequately.

## Methods

### Settings

The study was conducted in the large, central Indian province of Madhya Pradesh (MP). Over two-thirds of MP’s 72 million population is rural [15]. A third of all inhabitants live below the poverty line [16]. Infant mortality stands at 67 per 1,000 births, which is the highest in India [17]. Based on two sub-national surveys, point estimates for MMR in MP currently stand between 230[7] and 277 [18] maternal deaths per 100,000 births. The public sector is the dominant provider of obstetric services in the province. The private health sector is small, concentrated in urban areas, and unaffordable for the majority. In Madhya Pradesh, the JSY program has functioned largely through public sector facilities. The public health system has a three-tiered network of facilities: each district in the province has a top-level district hospital (DH) which is a tertiary-level hospital handling cases arriving directly or referred from community health centers (CHCs) that are secondary-care facilities within districts. CHCs in turn receive cases arriving directly or referred from primary-care centers (PHCs) in the outskirts of the district. All tiers of the public sector are accredited facilities for the JSY program. All pregnant women in MP are eligible for participation. The JSY provides a cash transfer of USD 31 to rural mothers and USD 22 to urban mothers.

### Study districts

Districts are administrative units within a province. Each district has a population between 1–1.5 million. Of the 50 districts in MP, three heterogeneous districts were selected for this study based on their geographic location and differing socio-economic levels of development (as indicated by human development indices). Table 1 provides selected indicators of study districts compared to provincial and national averages at the time of district selection.

**Table 1:** Selected characteristics of study districts compared to MP and India

**Health/development indicator**	**District 1**	**District 2**	**District 3**	**MP**	**India**
Maternal mortality ratio^1^	435	369	268	310	212
Neonatal mortality ratio^1^	49	66	33	44	33
Literacy rate (%)^2^	68	66	73	71	74
Institutional delivery (%)^3^	58	72	81	76	78
Human development index^4^	0.5	0.4	0.6		

Source- ^1^Annual Health Survey and ^2^Census of India 2011, Registrar General of India, Government of India; ^3^Family welfare statistics 2011, Government of India; ^4^Human Development Indices of districts of MP 2001.

### Study participants

In the three selected districts, all public facilities conducting 10 or more deliveries a month in the six month period prior to initiation of the survey (Sept 2012 – Feb 2013) were included in this study. All nurses who are routinely deployed on duty as frontline delivery-room nurses, or as their supervisors in the selected facilities, were invited to participate in the study by responding to a vignette-based survey. These in-service nurses hold either a basic 18-month Auxiliary Nurse Midwife (ANM) qualification, three years training in General Nursing and Midwifery (GNM), or four years training to qualify with a Bachelor of Science in Nursing (BSc).

### Study design

Cross-sectional survey based on written case vignettes.

### Definition of competence

**Figure 1:**
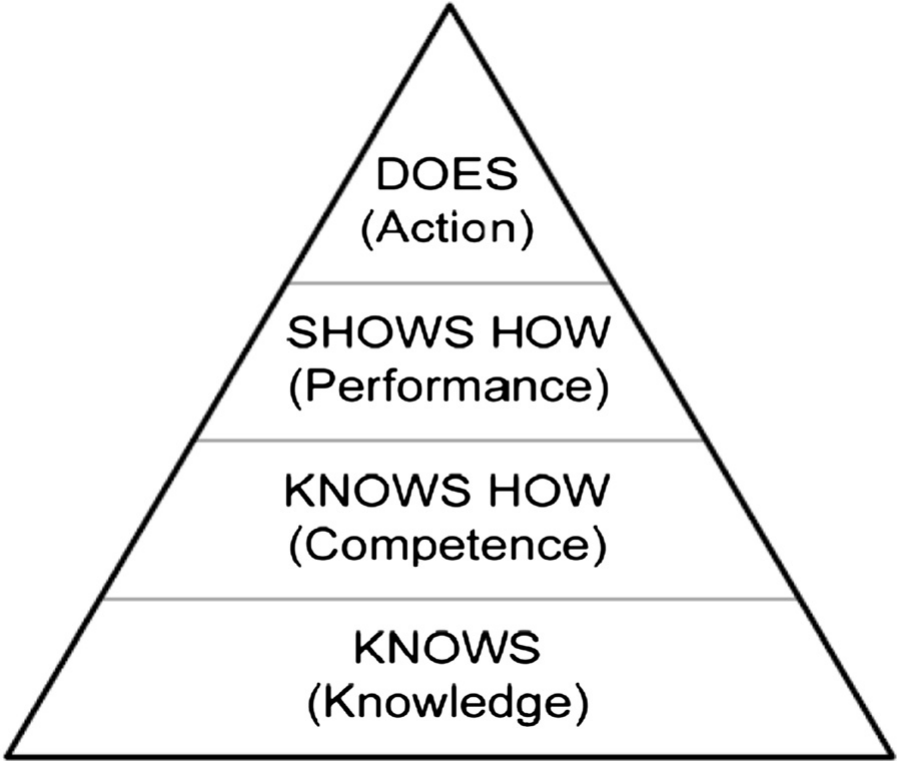
**Framework for clinical assessment by Miller **[19]**.**

We used the framework provided by Miller for assessment of clinical competence [19] (Figure 1). Miller portrays competence as a stage that follows knowledge acquisition and leads to performance. We thus defined competence as the ability to apply knowledge in concrete situations.

### Development of vignettes

The model for construction of case vignettes proposed by Heverly et al. was used to guide the vignette development process [20]. There were four main steps in the development of vignettes: identifying study factors, generating vignette components, constructing the vignettes, and validation as described below.

1. Identifying the main factor of interest for the study: Given the criticality of the competence of birth attendants in providing EmOC, obstetric complications (and their management) were the main factors to be studied using vignettes. Hemorrhage and eclampsia were chosen as the two complications, as these are the leading causes of maternal deaths in the study province [21]. Two vignettes were developed for each condition; thus a total of four vignettes were developed – (i) ante-partum hemorrhage (APH), (ii) post-partum hemorrhage (PPH), (iii) ante-partum eclampsia occurring in a woman with pregnancy-induced hypertension detected during ante-natal care, and (iv) ante-partum eclampsia in a woman with no previous antenatal care. Each respondent was presented with one hemorrhage vignette and one eclampsia vignette.

2. Generating vignette components: The JSY program to promote institutional births was complemented by instituting Skilled Birth Attendant (SBA) training in 2005. SBA training is a three-week residential training for in-service, nurse-midwives. It involves hands-on training at tertiary-level hospitals that are designated training sites. It covers care during pregnancy and normal delivery and identification and management of life- threatening obstetric complications. The handbook for SBA training [22] produced by the Government of India was used as a knowledge base to develop the vignettes and the relevant questions. Content of the SBA training is part of the curriculum of basic nursing education (ANM and GNM) and forms the prescribed content for the in-service SBA training of nursing staff in the public sector. The handbook is in line with global standards of essential competencies for basic midwifery practice set by the International Confederation of Midwives 2010 [23].

3. Construction of vignettes: The vignettes were initially developed by the research team. (Vignettes are provided in Additional file 1). Each case description was followed by three to four unambiguous questions to be posed to the respondent nurse-midwives on initial assessment of the patient, then on diagnosis, and then on first-line care and advice. All questions were open ended.

4. Establishment of validity: Content validity was established by discussing the draft vignettes with three senior obstetricians. The obstetricians had experience of working in contexts similar to the study settings. They also ascertained that all vignettes had the same level of technical ease/difficulty. The vignettes were translated into the local language, Hindi. To assess the face validity of the prepared vignettes, we discussed these with delivery-room nurse respondents from all levels of facilities. A pilot test (n = 20) of the developed vignettes was undertaken in a neighboring district not included in this study.

### Scoring method

The standard responses to each of the questions following a vignette were developed in line with standard practices recommended by the SBA handbook. These were finalized in consultation with the same experts who assessed the vignettes for content validity. The relative importance of different tasks for assessment and or management of each complication was considered. For instance, measurement of blood pressure received a relatively higher score than looking for pallor in the eclampsia case, while in the PPH case, starting an IV fluid with Injection Oxytocin 20 IU was scored higher than administering alternate drugs. The maximum score for each vignette response was 10. The scoring scheme was also tested by using responses from the pilot study (Scoring scheme is provided in Additional file 2).

### Administering the vignette survey

A researcher first contacted the head nurse at each study facility and introduced herself/himself as being from the medical college and the project. She/he then requested to be introduced to nurses routinely posted on delivery-room duty. The researcher (with medical training) then met each nurse individually and built a rapport with her. The purpose of the study was explained to each nurse emphasizing that it was aimed to assess the average competence of nursing staff providing obstetric care in the JSY program, and was not in any way an individual assessment. Each respondent was then presented with the written vignettes while on the ward. She was asked to write her responses to the questions, so that they reflected the appropriate action to take when attending to such a patient as the vignette described. Each respondent received two vignettes; one vignette for each condition i.e. hemorrhage and eclampsia. Each vignette was administered in an unfolding, sequential manner. The case scenario with questions about clinical assessment of the case was presented first. On obtaining the response sheet, additional information from clinical examination of the case, with short questions on provisional diagnosis and first-line care, was provided. On average, respondents took 20 minutes to complete the responses.

### Scoring

The first two authors independently scored the responses. The total possible score for an individual respondent was 20. While scoring, a record of incorrect responses was maintained.

### Ethical issues

Researchers spent time to build good rapport with potential participants. After the introduction of the study, the potential participants were given an opportunity to seek answers to any relevant questions. Participation was voluntary, and no incentives to participate were provided. Consent was obtained prior to participation. The responses were anonymous. The response sheets were strictly accessible only to study team members. Approval to conduct this study was granted by the Institutional Ethics Committee of the R D Gardi Medical College, Ujjain, India (Approval No. 245).

### Analysis

Data was entered initially into Excel spreadsheets. STATA 10 was used for analysis. Scores were presented using descriptive statistics including medians, ranges, and histograms. Differences by level of facilities, qualification, and districts were tested by the Kruskal Wallis test, while the Mann Whitney test was used for differences in competence by age, experience, SBA training, and average number of deliveries performed. Agreement between competence scores by the two raters was assessed using the reliability coefficient.

All items were scored to arrive at the overall competence score. Having arrived at an overall competence score for each participant, we further aimed to assess levels of competence in domains. For this, responses were categorized into three main domains; namely initial assessment, diagnosis, and first-line care. These domains were in line with the questions that followed the vignette presentation. To determine participants’ competence in a domain, a few items, which were considered critical, were selected from the list of items on the scoring scheme. The selection of these items was done through consultation with an expert. A participant was considered competent in a particular domain if each of the items identified as critical to this domain were mentioned in her response; those mentioning some or none of the critical items were considered incompetent in that domain.

## Results

1. Characteristics of study facilities and participants: The study districts had 73 facilities eligible for the study and all were included. The facilities, numbered by level of care and number of participants, are detailed in Table 2 below.

Participants: Of the 256 nurses who were eligible to participate, 91% were engaged in the study. The 21 non-participants were away from the facility on training sessions or on leave. Only one potential participant refused.

**Figure 2:**
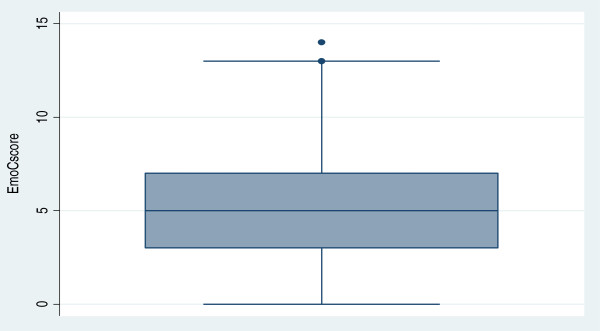
Competence scores (maximum score = 20).

Among participants, 66% (n = 153) were ANMs, 28% (n = 66) were GNMs, while 6% (n = 14) had a Bachelor of Nursing degree. Two third of ANMs were at primary-care facilities, though some were also posted at higher-level facilities. GNMs (95%) and BSc nurses (92%) were mostly at secondary and tertiary-care facilities. This distribution was similar across the three districts. The characteristics of participants are described in Table 3 below.2. The competence scores ranged between 0 and 14 (out of 20) with a mean score of 5.4 (27%) (median score = 5(25%)). The box plot of competence scores in Figure 2 reveals that 75% of the participants scored below 7 which translates to below 35% of the total possible score.

**Figure 3:**
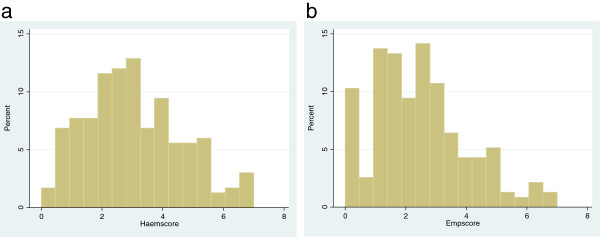
Histogram showing distribution of scores for Haemorrhage (3a) and for Eclampsia (3b); (maximum score = 10).

The competence score was marginally higher (yet the median was always below 35% of the total) among respondents with SBA training, those with GNM qualification, those who had conducted a higher number of deliveries, those from tertiary-level facilities, and those in a developed district, than their counterparts (Table 4). The scores did not vary by respondents’ age and years of experience in maternity. However, despite there being statistically significant differences in the scores between subgroups, it is clear that these differences have little clinical implication, as the scores were low overall, rarely exceeding 35% of the maximum score.The median scores for the vignettes on hemorrhage and eclampsia were 3 and 2.5 respectively on a maximum of 10 each. The scores were concentrated to the left (lower end) for both these conditions as seen in distributions in the histograms (Figure 3).

Although about two thirds of respondents recognized the need to refer the hemorrhage case, only a fifth mentioned the essential elements of stabilization prior to referral.

3. Competence scores for domains of competence: For a participant to be considered competent in a particular domain, the items that were considered as essential to be mentioned in the response are presented in Table 5.

**Figure 4:**
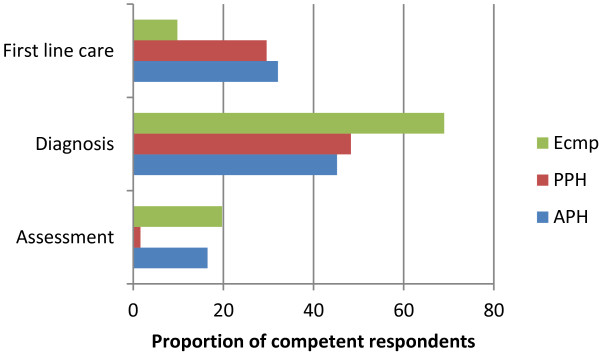
Proportion of competent respondents domain wise and by complication type.

Assessing the competence in domains, by applying the criteria described in Table 5 to the responses, only 14% of participants were found to be competent at initial assessment of the studied complications.Although 58% were able to arrive at a correct clinical diagnosis, only 20% were competent at providing appropriate first-line care for complications. The proportion of respondents competent in each domain, by complication, is presented in Figure 4. Competence was poorest for PPH assessment, while first-line care was poorest for eclampsia, in contrast to the ability to diagnose it.

Participants competent at diagnosis were not all competent at management of the complication. Of the respondents competent at diagnosis, those who were also competent at first-line care thereof were 44% for APH, 39% for PPH, and 13% for eclampsia.

4. Other responses: Apart from the above competence scores, with reference to the SBA standards, a list was generated of the other incorrect and less relevant responses that were not part of the scoring scheme (Table 6). These responses reflected participants understanding of the case and revealed what participants considered appropriate.

5. Inter-rater reliability of the scores was determined using intraclass correlation. The reliability coefficient obtained was 0.97 (95% CI: 0.95-0.99) indicating strong agreement.

**Table 2:** Distribution of study facilities and respondents by districts

**Facilities/districts**	**District 1**	**District 2**	**District 3**	**Total facilities**	**Number of participants at each level of care**
Primary care	20	21	12	53	97
Secondary care	6	5	6	17	99
Tertiary care	1	1	1	3	37
Total facilities	27	27	19	73	
Number of participants from each district/total	73	94	66		233

**Table 3:** Participant characteristics

**Characteristic**	**Median (range)/percentage**
Age	36 (21–65) years
Total experience	10 (0.5-40) years
Maternity experience	5( 0–39) years
Average deliveries per month	15 (0–300)
Proportion SBA trained	56%
Proportion Females	100%

**Table 4:** Differences in competence score by participant characteristics

**Characteristic**	**Category**	**Median score (% max score)**	**P value**
Age	≤ 35 years	5.5 (27.5)	0.71
	>35 years	5 (25)	
Average deliveries conducted	≤ 30/month	4 (20)	0.00
	>30/month	6 (30)	
Maternity experience	≤5 years	5.5 (27.5)	0.79
	>5 years	5 (25)	
**SBA training**	**No**	4 (20)	0.00
	**Yes**	5.5 (27.5)	
Qualification	ANM	4 (20)	0.00
	B Sc	6 (30)	
	GNM	7 (35)	
Facility level	Primary	3.5 (17.5)	0.00
	Secondary	6 (30 )	
	Tertiary	7 (35)	
District	District 2 (HDI* 0.4)	4.5 (22.5)	0.00
	District 1 (HDI 0.5)	4.5 (22.5)	
	District 3 (HDI 0.6)	7 (35)	

*HDI-human development index.

**Table 5:** Constituents of domains of competence by complication type – critical elements necessary to be mentioned in the response to be considered competent in each domain

**Domain/ complication**	**APH**	**PPH**	**Eclampsia**
Initial Assessment	Pulse, Blood Pressure, Per Vaginum exam not to be conducted	Pulse, Blood pressure, Estimation of vaginal bleeding	Blood pressure, Urine examination for albumin
		Abdominal examination	
Diagnosis	APH/Placenta previa	PPH/Atonic PPH/Haemorrhagic shock	Eclampsia or Severe Pre eclampsia
First line care	IV fluids	IV fluids	Injection Magnesium sulphate, in right dose (5 gm in each buttock) and route (deep IM)
	Referral/consults doctor	Either adding an uterotonic drug or mentioning uterine massage	

**Table 6:** Other responses for complications

**Domain**	**APH**	**PPH**	**Eclampsia**
Initial assessment & diagnosis	Per vaginum examination (34%)	Exploring the uterus for blood clots (39%)	Weight (4%)
			Swelling on body parts related to severe anemia (8%), blurred vision related to night blindness (1%)
			Diagnosed eclampsia as pre eclampsia or pregnancy induced hypertension (26%)
First line management	Antibiotic (12%)	Alternate drugs mentioned:	Diazepam (5%)
		Ergometrine (34%)	
	Iron folic acid (11%)	Misoprostol (15%)	Diazepam with MgSO4 (3%)
	Misoprostol and ergometrine (<1%)	Unindicated drugs mentioned:	Antibiotics (5%)
		Injection Tranexamic acid (1%),	Anti hypertensive (6%)
		Antibiotics (21%)	
		Ironfolicacid (11%) Calcium and multivitamin (9%)	
		Vaginal packing (1%)	
Advice	Dietary advice and regular ANC (12%)	Dietary advice and regular ANC (20%)	Regular ANC (3% )
	Perceived APH is caused by sexual activity or lifting weight; hence advised bed rest	New born care, Early breast feeding	
		And Family planning (1%)	

## Discussion

To the best of our knowledge, this is the first study to assess competence in management of complications under the JSY. The poor levels of competence that this study has found could in part explain the slowness of decline in maternal mortality despite a successful institutional-birth promotion under the JSY program in India. Hulton et al. [24] in their framework for quality of maternal health, specify the proportion of trained staff who recall the signs and key procedures in providing first-line management for hemorrhage and hypertensive disorders as an indicator of quality. The poor competence at assessing and initiating treatment for these conditions found in this study raises questions about the appropriateness and safety of obstetric care in the JSY program in MP. The study identifies priority remedial action areas for stakeholders interested in maternal mortality reduction in general, and specifically under the JSY program.

### Poor competence in initial life-saving management of complications

Our findings of poor competence scores indicate a low possibility of women receiving life-saving EmOC under the JSY program in MP.A high proportion of respondents made a decision to refer the patient, although they were not competent at providing first-line care. This indicates a low possibility of proper stabilization before referral. It is known that if women with complications are referred without proper stabilization, they risk death enroute to, or at, higher-level facilities as has been reported by other Indian studies [25,26].

The higher proportion of respondents competent at making a diagnosis (58%), compared to those competent at initial assessment (14%), suggests respondents possibly guessed the diagnosis, and did not base it on a judgment from clinical assessment of the patient. In the case scenario of APH, 34% of nurses mentioned they would do a per vaginal (PV) exam as part of their initial assessment. These findings reveal that apart from poor competence at conducting proper assessment, the staff did not perceive this practice as being potentially harmful. Findings show participants lacked clinical understanding of a condition like hemorrhage. Participants seem to have assumed hemorrhage to have an infectious etiology, and hence antibiotics were frequently mentioned in the case of hemorrhage. The frequent mention of un-indicated drugs and those not routinely available in public supplies could imply a waste of critical time (spent in procuring and administering these) in the face of a life-threatening emergency and a waste of resources for families and the health system.

The poor levels of competence of nurse-midwives in our study are in consonance with another recent study from MP. The study investigated maternal deaths at a tertiary hospital and reported a lack of competent EmOC resulting in preventable maternal deaths [27]. Although there have been no other reports from studies specifically of competence for EmOC provision in India, a study by Das et al. [28] showed poor competence of primary care providers, in rural MP, for general practice in adult and pediatric conditions. Studies from other contexts dealing with high maternal mortality also point to gaps in provider knowledge and skills. For instance Partamin et al. [29] found the SBAs in Afghanistan were weak at managing common maternal emergencies as assessed from performance on anatomical models. Ijadunaola et al. [30] reported 91% of staff in Nigeria had poor knowledge of the EmOC concept while Ariff et al. [31] found all levels of staff in Pakistan performed below the competency levels for maternal and neonatal health knowledge and skills. Harvey et al. [32] found large gaps between standards and provider competence to manage selected obstetric complications in Benin, Ecuador, Jamaica, and Rwanda. Interestingly, maternal health workers in Nepal [33] demonstrated acceptable knowledge and skills to function as community-level skilled attendants; however this assessment was likely to be less standardized.

### Non perception of ‘emergency’ in EmOC

The responses strongly indicated that nurses possibly failed to perceive the emergency in the situation. Their responses on advice to attendants largely included family planning, ferrous-sulfate tablets, and an iron rich diet, while very few mentioned explaining the urgency of the situation or possible need of blood transfusion. The nurses seemed to identify themselves more as routine ante-natal care providers and promoters of population-stabilization programs rather than skilled birth attendants providing life-saving first-line EmOC.

### Poor competence- a re-look at training

The poor competence as demonstrated by our results suggests a need to re-look at both pre-service and in-service training for nurse midwives. Pre- service education is provided in public as well as private nursing schools in MP as in other Indian provinces. Though norms for nursing education are defined, there seems to be a significant deficit in the quality of training provided. In-service SBA training was implemented along with the JSY to ensure technical competence at EmOC while increasing access to institutional delivery care. Hulton et al. [24] include training opportunities for staff as indicators of human-resource quality. However the competence levels are only marginally better, though still very low, among those with SBA training. This questions the effectiveness of SBA training and calls for a need to understand and correct the failure to produce competencies. Ensuring adequate competence of trainers who deliver this training is an important consideration. Also, when nurse midwives return to their work settings after skills- building training such as SBA training, competent supportive supervision is essential to practice these skills effectively and so improve health outcomes. Currently, co-workers in a supervisory capacity, also lack the required competence; the other cadre of trained workers is the medical doctors who often perform a more administrative rather than clinical role. Some attend a few deliveries but normally do not provide clinical training and supervision to nurse midwives. A continuous midwifery-education program would no doubt be useful, provided appropriate training methods are actually used, with adequate opportunities for well- supervised, practical training. Use of innovative training aids, for instance low-cost birth simulators that have proven to be successful in training nurse/midwives in obstetric emergencies in other low resource contexts are promising options to be considered [34].

### Competence of staff at providing EmOC critical for JSY program success

The Indian situation with regard to institutional delivery care and maternal mortality reduction resembles that reported from the Dominican Republic in 2000, where maternal mortality remained high despite there being near universal access to institutional care [35]. Although uncertainties around the measurement of MMR, as well as the proportion of institutional births, cannot be denied, the chances of improving maternal outcomes soon are small if competence at EmOC provision among front-line staff under the JSY is not improved, especially in high maternal mortality provinces. The Government of India, in its recent recognition of the need to have staff competent at providing quality midwifery services, has released operational guidelines [36] for strengthening pre-service midwifery education. This is a positive step, though more thorough consideration of the issues raised above is required.

#### Methodological discussion

Our experience with use of vignettes is in agreement with several authors who suggest vignettes are a valid method to assess quality on a large scale [37,38] and for assessing intra-partum decision making [39]. Vignettes are known to be ideal for situations, as in our study, that require keeping patient variables constant [40,41]. Vignettes have been used in studies that evaluate quality of clinical practice in real life settings and for comparisons across nations [42]. Vignettes have advantages over alternate methods of assessing competence. For instance, properly trained, standardized patients are expensive, intrusive, and their use can be impractical, especially to assess competence in emergency situations and in low-resource settings. Peabody et al. [43] conducted a large validation study of vignettes and found vignettes to be better measurements of quality than medical record abstraction. They report vignettes to be robust in the measurement of clinical quality across different patient conditions, different sites, case complexities, and levels of physician training. Although use of vignettes is popular in midwifery, to the best of our knowledge, they have not been used before to assess competence in EmOC. We have demonstrated this use and its feasibility in the Indian context.

This study draws strength from using written vignettes. We were thus able to overcome possible limitations of using simulated clients, or observations, for assessment of competence at EmOC. We were also able to score responses objectively and present complicated cases to all respondents, adjust for case mix, and allow comparison of results across facilities.

It could be argued that actual practices are different from the competence that vignettes assess. Though this is a possibility, it is competence that is translated into practice, and hence competence is likely to reflect practice. Actual assessment of performance/skill could have been done by direct observation. However this was not a feasible approach in the study context. Although vignettes do not measure actual practice, they measure abilities to do so. Studies show that competence assessed by vignettes tends to be a more optimistic assessment than that assessed using standardized patients in actual clinical settings [44] and from direct observation [45]. Studies suggest that while vignettes are a useful quality evaluation tool, they are not complete when used in isolation and are not replacements for direct observation. Our findings need to be interpreted bearing this in mind.

The inability of some respondents to express themselves in writing, even when they have made the right clinical judgment mentally, could result in an underestimation of staff competence. However, with this in mind, we ensured the responses required no more writing effort than is required in routine obstetric nursing practice, and so the possibility for such bias is remote. The invisible process of judgment and decision making is challenging to study; Heverly et al. [20] conclude that written vignettes, using experimentally controlled stimuli to elicit judgment, are better suited for such study than observations or interviews.

##### Generalizability

Our study settings are the public sector facilities in MP. There could be limitations to the generalizability of the findings across the country. Although findings could vary between different provinces in India, the situation with regard to competence in the nine high maternal mortality provinces (that are the focus of the JSY) is possibly somewhat similar to that found in our study in MP, owing to similarities with regard to levels of socio- economic development, education, health system functioning, and implementation of the JSY.

## Conclusions

Institutional births under the JSY program do not imply access to competent EmOC. Given the low levels of competence of nurse midwives in the JSY program at EmOC provision, the proportion of institutional deliveries may not appropriately reflect progress towards the JSY goal of maternal mortality reduction. Raising competence in EmOC provision is a key opportunity to translate the large gains in coverage of institutional delivery services under the JSY into reductions in maternal mortality in MP.

Current pre-service and in-service training has not resulted in building the required competence of nurse midwives. Improvement in the quality of nursing and midwifery education is a crucial step to create a technically competent workforce rather than merely qualified personnel. Adoption of training methods that build the confidence and competence to provide life-saving care is important for the effective functioning of nurse midwives in peripheral facilities. Supportive supervision during training, as well as on the job, could be an important step to bridge the current gaps in competence.

## Competing interests

The authors declare that they have no competing interests.

## Authors’ contributions

SC conceptualized and designed the study, acquired and analyzed data, and wrote the manuscript. SU collected the data and participated in analysis and writing. ADC participated in study design, interpretation of results, and critically revised the manuscript. All authors read and approved the final version.

## Pre-publication history

The pre-publication history for this paper can be accessed here:

http://www.biomedcentral.com/1471-2393/14/174/prepub

## Supplementary Material

Additional file 1 Case vignettes. Click here for file

Additional file 2 Scoring scheme. Click here for file
