# Supplementary material for: Competence of birth attendants at providing emergency obstetric care under India’s JSY conditional cash transfer program for institutional delivery: an assessment using case vignettes in Madhya Pradesh province
Source: BMC Pregnancy Childbirth. 2014 May 24;14:174. doi: 10.1186/1471-2393-14-174 (PMC4075933; doi:10.1186/1471-2393-14-174)
Supplement: Additional file 1 — Case vignettes. [file 1471-2393-14-174-S1.docx]

**Vignette 1**

Sitabai is 22 years old. She is pregnant for the first time. She has just completed eight months of pregnancy and had no problems so far. She has come to your hospital with her husband and mother-in-law. She appears anxious and is sweating profusely. She tells you that since morning she has vaginal bleeding, it is bright red and she does not have any pain.

1. What examinations will you conduct to assess Sitabai’s condition? Which specific

examination would you avoid conducting?

On examination you find the following: Sitabai appears pale and her skin is cold. Pulse 110/min, BP -90/60 mm Hg, Temperature 36.8 deg Celsius, Respiratory rate- 32/min, 1 vaginal pad soaked in last 5 minutes, FHR- 110 b/min, Presentation is vertex and longitudinal lie.

2. What do you think is the probable diagnosis?

3. How would you manage the case?

4. What advise would you give to Sitabai’s husband?

**Vignette 2**

Sitabai is 20 years old. She had a full term pregnancy and delivered at home one and half hour ago. Her grandmother and the village dai conducted the delivery. They have brought Sitabai to your hospital because she is having heavy bleeding since delivery. Sitabai was in labour for 12 hours and had a normal delivery, the placenta was expelled 20 minutes after the birth of the baby.

1. What examinations will you conduct to assess Sitabai’s condition?

On examination you find the following: Sitabai appears pale and confused. Pulse 110/min, BP -80/60 mm Hg, Respiratory rate- 28/min, 1 vaginal pad soaked in last 5 minutes, On abdominal examination uterus is found to be soft and relaxed and there are no vaginal tears.

2. What do you think is the probable diagnosis?

3. How would you (as a nurse at the facility) manage the case?

4. What advice would you give to Sitabai’s husband?

**Vignette 3**

Arifa is 21 years old. She is pregnant for the first time. She is taking regular ANC at a PHC. The doctor has given her tablets in the seventh month and asked her to take it every day as she had high blood pressure and swelling on the body. She is now in the 9^th^ month of pregnancy. Arifa complained of severe headache and blurred vision this morning .Her husband and mother-in-law have brought her to your hospital today and they tell you that she had a convulsion on the way to the hospital.

1. What examinations will you conduct to assess Arifa’s condition?

On examination you find the following: Arifa’s Pulse 110/min, BP -160/100 mm Hg, Respiratory rate- 24/min, There is no pallor, On abdominal examination lie is found to be longitudinal and presentation is cephalic, FHR 110 b/min.

2. What do you think is the probable diagnosis?

3. What first line care would you provide to Arifa before doctors help is received?

**Vignette 4**

Arifa is 24 years old. She is pregnant for the first time. She has never taken ANC. Looking at her you feel she must be about 36 weeks pregnant. Her husband and mother-in-law have brought her to your hospital and they tell you that she had a convulsion on the way to the hospital. They also tell you that she was complaining of blurred vision and headache in the morning.

1. What examinations will you conduct to assess Arifa’s condition?

On examination you find the following: Arifa’s Pulse 110/min, BP -160/100 mm Hg, Respiratory rate- 24/min, There is no pallor. On abdominal examination lie is found to be longitudinal and presentation is cephalic, FHR 110 b/min.

2. What do you think is the probable diagnosis?

3. What first line care would you provide before doctors help is received?
