# Supplementary material for: Competence of birth attendants at providing emergency obstetric care under India’s JSY conditional cash transfer program for institutional delivery: an assessment using case vignettes in Madhya Pradesh province
Source: BMC Pregnancy Childbirth. 2014 May 24;14:174. doi: 10.1186/1471-2393-14-174 (PMC4075933; doi:10.1186/1471-2393-14-174)
Supplement: Additional file 2 — Scoring scheme. [file 1471-2393-14-174-S2.docx]

**Scheme for scoring responses to vignette 1**

| **S.No** | **Criteria** | **Score** |
| --- | --- | --- |
|  | In initial assessment |  |
| 1.1 | Mentions pulse | 01 |
| 1.2 | Mentions BP | 01 |
| 1.3 | Mentions temperature | 0.5 |
| 1.4 | Mentions looking for pallor  (or specifies looking at eyes,tounge or nails or mentions Hb test) | 0.5 |
| 1.5 | Mentions Respiratory rate | 0.5 |
| 1.6 | Mentions estimation of vaginal bleeding (or counting pads soaked or asking the patient about the amount of bleeding ) | 0.5 |
| 1.7 | Mentions PV not to be conducted | 01 |
| 2. | Probable Diagnosis- Either APH (mentions either acronym or full form) or shock due to bleeding or placenta praevia | 01 |
| 3. | In case management: |  |
| 3.1 | Mentions starting IV RL 500ml | 01 |
| 3.2 | Mentions starting IV @60 dps/min or mentions fast drip/full speed | 01 |
| 3.3 | Mentions the following:  Respondent at NonCEmoc facility-PHC/CHC-referral to higher centre/emergency referral  Respondent at CEmOC -higher level facility – calls doctor/specialist | 01 |
| 4 | Advise to husband |  |
| 4.1 | Mentions that patient may require blood transfusion | 0.5 |
| 4.2 | Mentions should cover patient (in hospital/during transport | 0.5 |
|  | **Maximum score** | **10** |

**Scheme for scoring responses to case vignette 2**

| **S.No** | **Criteria** | **Score** |
| --- | --- | --- |
|  | **In initial assessment** |  |
| 1.1 | Mentions pulse | 0.5 |
| 1.2 | MentionsBP | 0.5 |
| 1.3 | Mentions temperature | 0.5 |
| 1.4 | Mentions looking for pallor  (or specifies looking at eyes,tounge or nails or mentions Hb test) | 0.5 |
| 1.5 | Mentions Respiratory rate | 0.5 |
| 1.6 | Mentions estimation of vaginal bleeding (or counting pads soaked or asking the patient about the amount of bleeding ) | 0.5 |
| 1.7 | Mentions abdominal examination/looking for contraction of uterus | 01 |
| 1.8 | Mentions looking for vaginal/perineal tears  ( Score 0.5 if only PV is mentioned and looking for tears is not mentioned) | 01 |
| 2. | Probable Diagnosis- Either PPH (mentions either acronym or full form) or atonic PPH or shock due to postpartum bleeding | 01 |
| 3. | In case management: |  |
| 3.1 | Mentions starting IV RL 500ml | 0.5 |
| 3.2 | Mentions adding to the IV fluid injection Oxytocin 20IU (score o.5 if injection Oxytocin is mentioned but 20 IU is not mentioned or if injection Methergin or Misoprostol is mentioned or Oxytocin IM is mentioned) | 01 |
| 3.3 | Mentions starting IV @60 dps/min or mentions fast drip/full speed | 0.5 |
| 3.4 | Mentions uterine massage | 01 |
| 3.5 | Mentions the following:  Respondent at NonCEmoc facility-PHC/CHC-referral to higher centre/emergency referral  Respondent at CEmOC -higher level facility – calls doctor/specialist | 0.5 |
| 4 | Advise to grandmother | 0.5 |
| 4.1 | Mentions either of the following   - that patient may require blood transfusion - should cover patient during transport /explains urgency |  |
|  | **Maximum score** | **10** |

**Scheme for scoring responses to case vignettes 3 and 4:**

| **S.No** | **Criteria** | **Score** |
| --- | --- | --- |
|  | **In initial assessment** |  |
| 1.1 | Mentions pulse | 0.5 |
| 1.2 | Mentions BP  (Score 0.5 if recognises high BP but does not specify examination) | 01 |
| 1.3 | Mentions temperature | 0.5 |
| 1.4 | Mentions looking for pallor  (or specifies looking at eyes,tounge or nails) | 0.5 |
| 1.5 | Mentions Respiratory rate | 0.5 |
| 1.6 | Mentions estimation of urine output or bladder catheterisation | 01 |
| 1.7 | Mentions urine examination /looking for proteins/albumin in urine | 01 |
| 2. | Probable Diagnosis- Eclampsia | 01 |
| 3. | In case management: |  |
| 3.1 | Mentions suction of mouth | 01 |
| 3.2 | Mentions placing mouth gag | 0.5 |
| 3.3 | Mentions positioning in left lateral position | 0.5 |
| 3.4 | Mentions starting/giving Magnesium sulphate injection | 01 |
| 3.5 | Mentions dose of Magnesium sulphate- 10 ml (or 5gm or 5 ampoules) in each buttock or 20ml (or 10 ampoules) in total | 0.5 |
| 3.6 | Mentions giving injection deep IM in each buttock | 0.5 |
|  | **Maximum score** | **10** |

Acronyms : BP- Blood pressure, PV- Per vaginum examination, Hb- Haemoglobin, APH- Ante partum haemorrhage, PPH- Post partum haemorrhage ,IM- Intra muscular,IV- Intravenous,Inj- Injection CEmOC- Comprehensive emergency obstetric care, PHC-Primary Health Centre, CHC-Community Health Centre
